# Synthesis and antimicrobial effects of silver nanoparticles produced by chemical reduction method

**Published:** 2010

**Authors:** S. kheybari, N. Samadi, S.V. Hosseini, A. Fazeli, M.R. Fazeli

**Affiliations:** 1 Department of Drug and Food Control and Pharmaceutical Quality Assurance Research Center, Faculty of Pharmacy, Tehran University of Medical Sciences, Tehran; 2 Biotechnology Group, Department of Chemical Engineering, Tarbiat Modares University, Tehran, Iran

**Keywords:** Nanoparticles, Silver, Ethylene glycol, Glucose, Colloids, Reduction method

## Abstract

**Background and the purpose of the study:**

The most prominent nanoparticles for medical uses are nanosilver particles which are famous for their high anti-microbial activity. Silver ion has been known as a metal ion that exhibit anti-mold, anti-microbial and anti-algal properties for a long time. In particular, it is widely used as silver nitrate aqueous solution which has disinfecting and sterilizing actions. The purpose of this study was to evaluate the antimicrobial activity as well as physical properties of the silver nanoparticles prepared by chemical reduction method.

**Methods:**

Silver nanoparticles (NPs) were prepared by reduction of silver nitrate in the presence of a reducing agent and also poly [*N*-vinylpyrolidone] (PVP) as a stabilizer. Two kinds of NPs were synthesized by ethylene glycol (EG) and glucose as reducing agent. The nanostructure and particle size of silver NPs were confirmed by scanning electron microscopy (SEM) and laser particle analyzer (LPA). The formations of the silver NPs were monitored using ultraviolet- visible spectroscopy. The anti-bacterial activity of silver NPs were assessed by determination of their minimum inhibitory concentrations (MIC) against the Gram positive (*Staphylococcus aureus* and *Staphylococcus epidermidis*) as well as Gram-negative (*Escherichia coli* and *Pseudomonas aeruginosa*) bacteria.

**Results and Conclusion:**

The silver nanoparticles were spherical with particle size between 10 to 250 nm. Analysis of the theoretical (Mie light scattering theory) and experimental results showed that the silver NPs in colloidal solution had a diameter of approximately 50 nm.

Both colloidal silver NPs showed high anti-bacterial activity against Gram positive and Gram negative bacteria. Glucose nanosilver colloids showed a shorter killing time against most of the tested bacteria which could be due to their nanostructures and uniform size distribution patterns.

## INTRODUCTION

The use of silver and other metal ions for their sustained anti-fungal, anti-bacterial and anti-viral effects have been practiced for a long time. Such effects are generally referred as oligodynamic action. Recent studies have focused on the synthesis of homogenous silver nanoparticles (NPs) and evaluation of their antimicrobial activities (1, 2). Silver ion has been known to be effective against a broad range of microorganisms. Silver NPs with higher surface to volume ratio compared to common metallic silver have shown better antimicrobial activity. Due to unique biological properties of silver NPs such as biocompatibility and anti-bacterial affinity they have been applied for various medical purposes such as implants, catheters, and healing of wounds (3, 4).

Synthesis of silver NPs has been of considerable interest during the past decades (5). A variety of methods have been reported for synthesis of metallic NPs. These include thermal decomposition, laser ablation, microwave irradiation, sonochemical, reverse micelles, salt reduction, radiolysis, solvothermal and electrochemical synthesize (6). However controlling the particle size and production of particles by an industrial scale is an important task of all methods. Chemical reduction of metal salts using various reducing agents in the presence of stabilizer is currently of interest for preparation of metal NPs (7, 8). However, it hitherto is difficult to realize a composition which provides a suitable oligodynamic antimicrobial effect and to be non- toxic to mammalian cells (less than 350 mcg/day allowed by Environmental Protection Agency as a maximum daily reference).

In the present study the synthesis of silver NPs by chemical reduction method using ethylene glycol and glucose as the reducing agents is described. Size distribution and antimicrobial activity of the silver nanocolloids are also compared.

## MATERIAL AND METHODS

### 

#### Materials

Silver nitrate (purity *>* 99%, Aldrich) was used as precursor in the preparation of silver NPs. Glucose and poly (N-vinylpyrrolidon) (PVP k30) were purchased from Merck. Ethylene glycol was purchased from Fisher (99.9%, HPLC grade).

#### Preparation of silver NPs

Two colloidal forms of silver NPs were prepared by one-step synthetic method using ethylene glycol and glucose as reducing agents. Uniform silver nanoparticles were obtained by reduction of silver nitrate at 50°-70°C under atmospheric pressure. Poly vinyl pyrrolidone (PVP) was used as stabilizer. Ethylene glycol silver NPs were synthesized by dissolving AgNO3 (157 mg) and PVP (5g) in 100 ml of 99.9% ethylene glycol (9). For the preparation of glucose silver NPs, AgNO3 (157 mg) and PVP (5g) were dissolved in 100 ml of 40% (w/w) of glucose syrup. In order to be confident that the reaction is complete and all the ionic silver have been converted to nanoparticles, 5ml of sodium chloride was added to the samples. Creation of turbidity in the reaction solution indicates the presence of ionic silver while a clear solution confirms completion of the reaction. Gravimetric method was utilized to measure the total nanosilver content of solutions. Nano silver particles were dissolved in 10% nitric oxide. Subsequently, sodium chloride (in excess) was added to the solution. The total amount of silver was determined by weighing the precipitated AgCl.

#### Characterization techniques

Size, morphology and composition of NPs were studied by scanning electron microscopy (SEM), laser particle analyzer (LPA) and ultraviolet-visible spectroscopy (UV-Vis). UV-Vis spectroscopy was performed using a Cecil CE 9200 super Aquarius Spectrophotometer. LPA was performed using a Zeta Sizer Malvern Nano-ZS. Size distribution of the particles was estimated using LPA images by measurement of diameters of at least 50 nanoparticles. SEM images were taken by “Zeiss- DSM 940 A” instrument.

#### Anti-bacterial determination

The antimicrobial activities of the NPs suspension were determined by measurement of their minimum inhibitory concentrations (MICs) using the standard micro dilution method (10). The bacterial strains used in this study were *Staphylococcus aureus* (ATCC 6538), *Staphylococcus epidermidis* (ATCC 12228), *Escherichia coli* (ATCC 8739) and *Pseudomonas aeruginosa* (ATCC 9027) which were provided by the Department of Drug and Food Control, Faculty of Pharmacy, Tehran University of Medical Sciences. Two-fold dilutions of silver NPs solutions and silver nitrate solution were prepared in Muller-Hinton broth in the concentration range of 2-512 ppm equivalent to pure silver content (11).

#### Bacterial death rate determination

The kinetics of death rates of the synthesized nanosilver particles as well as silver nitrate were determined by incubation of 107-108 CFU/ml of the individual bacteria in Muller-Hinton broth tubes.

## RESULTS AND DISCUSSION

### 

#### Physical characterization of the particles

**Figure 1:**
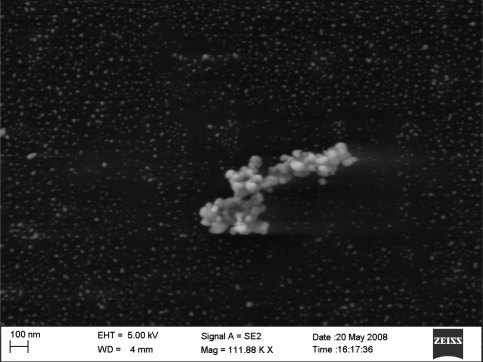
SEM image of silver nanoparticles in ethylene glycol silver colloid. Silver particles prepared from 1mM AgNO3 solution.

**Figure 2:**
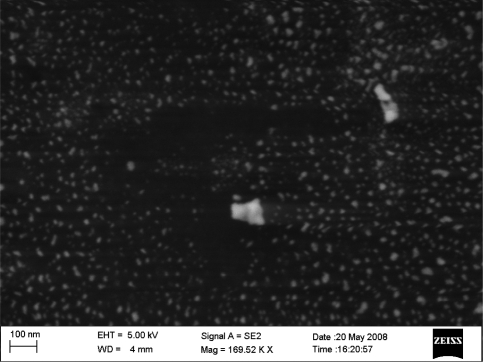
SEM image of silver nanoparticles in glucose silver colloid. Silver particles were from 1mM AgNO3 solution.

**Figure 3:**
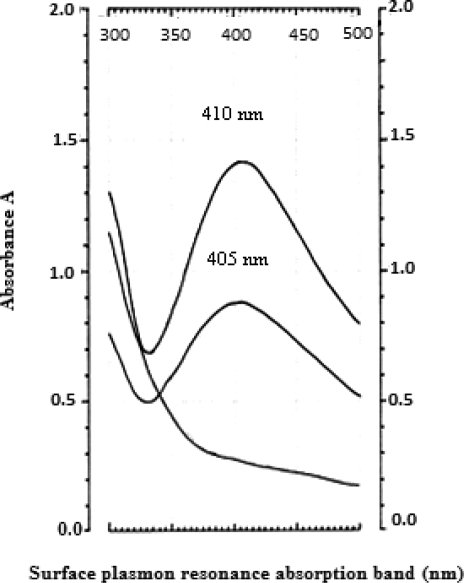
UV-Vis absorption spectrum of silver nanoparticles in ethylene glycol colloid (1), glucose colloid (2) and silver nitrate.

**Figure 4:**
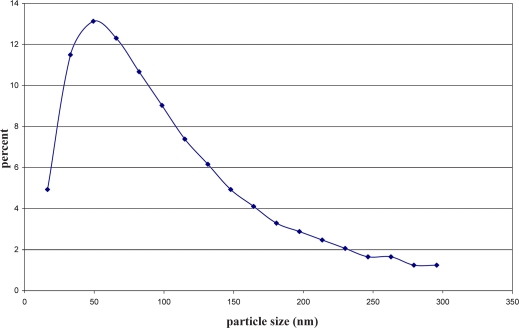
Particle size distribution of glucose nanosilver colloid using laser particle analyzer (Zeta Sizer Malvern Nano-ZS).

**Figure 5:**
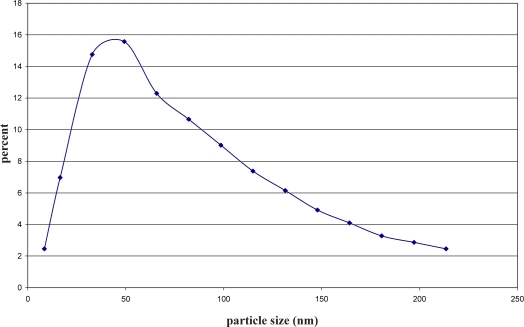
Particle size distribution of ethylene glycol nanosilver colloid using laser particle analyzer (Zeta Sizer Malvern Nano-ZS).

The SEM images of two types of silver NPs depicted in Figures 1 and 2 illustrates an uncontrolled growth of metallic particles in EG silver NPs. Figure 2 shows a SEM image of synthesised silver NPs by glucose which are spherical in shape and have a smooth surface morphology. It is also apparent that resulting NPs are more and less uniform in size and shape. The UV-Vis spectrum of the EG and synthesized NPs by glucose and also that of silver nitrate is illustrated in figure 3. Absorption between 400 – 450 nm is usually characteristic of silver NPs in the UV-Vis region (12). Figures 4 and 5 show size distributions of nanoparticles in two colloids which were determined by laser particle analyzer (Zeta Sizer Malvern Nano-ZS). While most of them were around 10-100 nm, smaller particles (8nm) were produced in glucose colloid rather than EG (15nm).

#### Anti-bacterial activity

Table 1 shows the MICs and MBCs of silver NPs and silver nitrate against the individual tested bacterial strains. These results tend to indicate that the EG silver NPs had higher anti-bacterial activity than glucose silver NPs.

**Table 1:** Minimum inhibitory and minimum bactericidal concentrations (MIC and MBC) of ethylene glycol and glucose silver colloids against some Gram positive and Gram negative bacteria compared with ionic silver.

MIC/MBC* (µg/ml)				
	
*S. aureus*	*S. epidermidis*	*P. aeruginosa*	*E. coli*	
18/20	15/22	9/12	11/12	Silver ion
20/20	10/16	7.5/12.5	10.5/20	EG silver NPs
28/28	20/28	22/36	19/21	Glucose silver NPs

* The figures quoted are means of at least three determinations.

**Figure 6:**
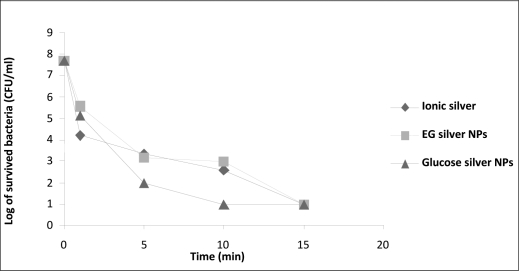
The kinetics of death rate of *S. aureus* in presence of ethylene glycol and glucose silver nanoparticles compared to silver nitrate.

**Figure 7:**
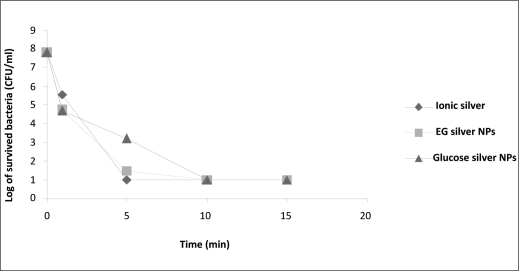
The kinetics of death rate of *S. aureus*, S. *epidermidis, P. aeruginosa* and *E. coli in* the presence of ethylene glycol and glucose silver nanoparticles compared to silver nitrate

**Figure 8:**
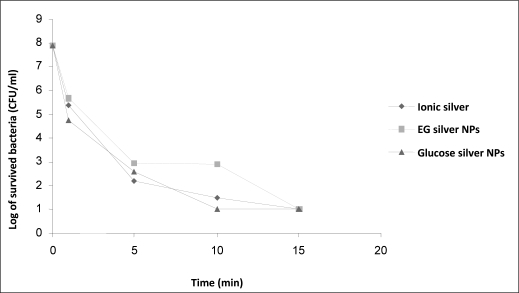
The kinetics of death rate of *P. aeruginosa* in the presence of ethylene glycol and glucose silver nanoparticles compared to silver nitrate.

**Figure 9:**
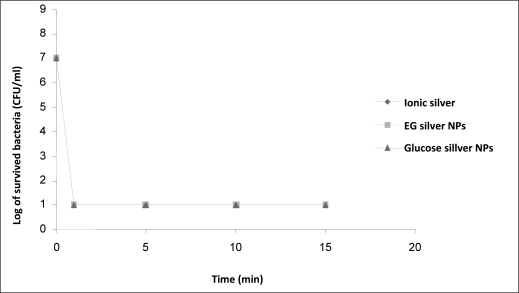
The kinetics of death rate of *E. coli* in the presence of ethylene glycol and glucose silver nanoparticles compared to silver nitrate.

The kinetics of death rate of different bacteria in solutions containing synthesized NPs by glucose and EG as well as silver nitrate are shown in figures 6–9. After 10 min the viable cells of *S. aureus* in glucose synthesized silver NPs solution showed 2 logs more than other solutions while the reduction rate for *S. epidermidis* was similar in all solutions. *E. coli* was the most sensitive to all silver compounds and the microbial count dropped to less than 10 CFU/ml after just one min. On the other hand *P. aeruginosa* was resistance to silver ions and glucose silver NPs. Synthesis of silver nanoparticles by chemical reduction is considered a cheap and simple method. In the current study poly vinyl pyrolidone (PVP) was used as a stabilizer of the silver NPs. PVP could also control the reduction rate of silver ions as well as aggregation of metal atoms (13). The reducing agent showed to have an important impact on the uniformity of the nanoparticles. The silver nanoparticles produced by using glucose as the reducing agent compared to those produced by ethylene glycol showed better uniformity.

The optical absorption spectra of metal nanoparticles are dominated by Surface Plasmon Resonances (SPR), which shift to longer wavelengths by increasing the particle size (14). Also small spherical NPs (<20nm) exhibit a single surface plasmon band (15) which is consistent with the results depicted in figure 3.

As shown in figure 3 the PVP-stabilized silver NPs solution, exhibited the surface plasmon resonance absorption band at 405 to 410 nm for glucose and EG colloids. The stability of silver NPs were monitored for 3 months which showed a SPR peak at the same wavelength. If nanoparticles in polymer have a narrow size distribution, the peak shape is symmetrical and the value of the full width at half- maximum (FWHM) is small (16). The silver colloids exhibited symmetric band and narrow FWHM for both colloids. Size distribution of the silver NPs were almost uniform and well dispersed (17).

The MICs of silver reported by Cho et. al were7.5µg/ml and 12.5 µg /ml for *P. aeruginosa* and *S. aureus*, respectively (18). Although the MICs of the current study against *P. aeruginosa* was close to the reported values. but those obtained for *S. aureus* were far beyond. Feng et.al, (19) reported an MIC of 14.1 mg of silver for *E. coli* which is in accordance with our results.

Figure 8 indicates that both NPs could present their bactericidal effects in less than 15 minutes against *P. aeruginosa* while Iroha et.al have recently shown that the colloidal silver could eradicate the same bacteria not less than 90 min (20). Glucose silver NPs showed better anti bactericidal effect on the tested bacteria in comparison to the EG particles. This could probably be due to the size distribution of particles in glucose which were smaller than the EG colloids.

The results of the present study suggest that silver NPs which were prepared using glucose as the reducing agent had a better particle uniformity and as a result superior anti-bacterial action compared to the nanosilver particles synthesized by using ethylene glycol. Although silver ions such as AgNO3 have a somewhat better antimicrobial effect but they are unstable in the presence of light or other radiation. Glucose nanosilver colloids are biologically compatible and have the potential to be used in medical and pharmaceutical applications due to their homologous size distribution and superior antimicrobial actions.
